# Research progress on surface antigen 1 (SAG1) of *Toxoplasma gondii*

**DOI:** 10.1186/1756-3305-7-180

**Published:** 2014-04-13

**Authors:** Yanhua Wang, Hong Yin

**Affiliations:** 1 State Key Laboratory of Veterinary Etiological Biology, Lanzhou Veterinary Research Institute, Chinese Academy of Agricultural Sciences, Lanzhou 730046, China

**Keywords:** *T. gondii*, Surface antigen 1, Gene structure, Invasion, Diagnosis, Vaccine

## Abstract

*Toxoplasma gondii* is an obligate intracellular parasitic protozoan that has a wide host range and causes a zoonotic parasitosis called toxoplasmosis. This infection causes significant morbidity, costs for care and loss of productivity and suffering. The most effective measures to minimize this parasite’s harm to patients are prompt diagnosis and treatment and preventing infection. A parasite surface antigen, SAG1, is considered an important antigen for the development of effective diagnostic tests or subunit vaccines. This review covers several aspects of this antigen, including its gene structure, contribution to host invasion, mechanisms of the immune responses and its applications for diagnosis and vaccine development. This significant progress on this antigen provides foundations for further development of more effective and precise approaches to diagnose toxoplasmosis in the clinic, and also have important implications for exploring novel measures to control toxoplasmosis in the near future.

## Review

### Background

*Toxoplasma gondii* is an obligate intracellular parasitic protozoan that is globally distributed, has a wide host range and causes zoonotic parasitosis [1,2]. *T. gondii* is an opportunistic infective agent and may cause death in individuals with compromised or suppressed immune functions, e.g., patients who suffer from AIDS (acquired immunodeficiency syndrome) or cancer or who have undergone organ transplantation. In addition, toxoplasmosis can have significant effects on reproduction because, if a pregnant woman is infected with *T. gondii*, maternal-fetal vertical transmission may occur and result in miscarriage, stillbirth or congenital defects or deformities (e.g., malformation, retardation, etc.) in infants regardless of the presence of clinical symptoms in the mother [3-5]. Moreover, the parasite also causes tremendous problems for livestock husbandry as the infection may lead to abortion and stillbirth in pregnant animals [6-8], especially cattle, goats and sheep [9], and high mortality rates in swine populations [10].

The tachyzoite is the key disease-causing form. During the acute stage of infection, tachyzoites activate a potent host immune response that eliminates most of the parasites. The surface of the tachyzoite is the main target of the host immune response. SAG1 is a major surface antigen involved in this process. Although it only accounts for 3-5% of total *T. gondii* protein, the majority of the antibodies are reactive against SAG1 during infection [11]. Various SAG1 preparations purified from the parasite [12,13], produced by recombinant systems in *Escherichia coli*[14] or yeast [15] and its constituent peptides [16-18], have all been demonstrated to stimulate host humoral and cellular immunity, thereby providing protection against toxoplasmosis [19]. As such, SAG1 features excellent antigenicity and immunogenicity and is valuable for both diagnosis and immunization. This article comprehensively evaluates the research progress on SAG1.

### Gene structure of SAG1

In 1980, Handman et al. [20] first identified SAG1 from *T. gondii* surface membrane antigens using a monoclonal antibody technique. Subsequently, Kasper et al. [21] applied mAb-affinity chromatography to isolate the protein. In 1988, Burg et al. [22] cloned the complete *T. gondii sag1* gene, which has a length of 1.1 kb, encodes 336 amino acids and yields a 30 kDa protein. In *T. gondii*, SAG1 anchors to the cell membrane by a glycosylphosphatidylinositol anchor. *Sag1* is a single-copy gene that does not have introns in the coding region or a TATA box in the promoter region. SAG1 transcription is governed by five 27-bp repeat sequences 35-190 bp upstream of the first two transcription start sites, and these repeat elements play a selective role in determining the transcription initiation site [23]. The repeat number of the conserved sequence is associated with the virulence of different *T. gondii* strains [24]. Specifically, highly virulent strains have five intact 27-bp repeats located 70 bp upstream of the transcription initiation site, whereas less virulent strains have only four repeats; additionally, the *sag1* expression level in virulent strains is over 4 times higher than that in avirulent strains. Sequence analysis of SAG1 cDNA revealed that 2 ATGs could function as the start codon and in turn generate signal sequences of 47 amino acids or 30 amino acids. Translation of *sag1* is generally accepted to begin with the second ATG codon, although both codons can be used to artificially start SAG1 synthesis. The nascent polypeptide resulting from a primary SAG1 mRNA transcript contains hydrophobic regions in both the N- and C-termini that are not present in most mature SAG1 proteins on the surface of *T. gondii*. However, the N-terminal hydrophobic region is believed to play a role in surface transport of the protein. After translocation, the signal peptide and the C-terminal hydrophobic region are cleaved and glycosylated, forming the mature SAG1 protein, which is anchored to the *T. gondii* cell surface. Although *sag1* sequences from different strains are highly similar, the gene contains polymorphisms, as evidenced by differences in SAG1 between the RH and ME49 strains at the amino acid level and the *sag1* gene sequence of ME49 strain being identical to that of the CEP strain except for a difference in the non-coding sequence [22,25,26].

### Roles of SAG1 in the invasion of *T. gondii* into host cells

*T. gondii* parasitizes not only phagocytes but also various non-phagocytic cells. Because this protozoan lacks several specific organelles, including flagella, cilia and pseudopodia, to facilitate invasion, multiple “receptor-ligand” interactions between the parasite and host cells exist. SAG1 has been demonstrated to be a crucial *T. gondii* ligand in promoting invasion of tachyzoites into host cells and allowing tachyzoite binding to host receptors. *In vitro* antibody neutralization experiments have revealed that a monoclonal anti-SAG1 antibody can partially block the invasion of tachyzoites into host cells [27-29]. Additionally, soluble SAG1 can directly bind to the host cell surface. The surface of SAG1 contains a groove to accommodate its dimerization. The groove region of the SAG1 dimer extends outwards from the cell surface and can effectively bind to its cognate ligands on the host cell surface [30]. Robinon et al. [31] demonstrated that soluble BSA-glucosamide blocks SAG1 attachment to MDBK (adult bovine kidney; Madin & Darby) cells using a competitive binding approach. Although anti-SAG1 antibodies and BSA-glucosamide inhibit tachyzoite attachment *in vitro*, it is not known whether their blocking effects are explicitly mediated through masking host cell binding domains on SAG1.

Conformational changes of SAG1 can influence *Toxoplasma* adherence to host cells [28]. For example, the *sag1* of the wild-type PLK strain is predicted to encode 9 cysteine residues, whereas the mutant strains PTgA or PTgC harbor mutations at the cysteine sites that cause protein misfolding and in turn diminish *T. gondii* adherence to human fibroblasts by 26% or 39%. *T. gondii* invasion into host cells is dependent on multiple factors. Two of these factors, SAG2 and SAG3, are in the vicinity of SAG1 on the *T. gondii* cell membrane and can assist SAG1 in facilitating rapid parasite invasion. Similar to *sag1, sag2* is also a member of a gene family, and these two gene families share weak homology. Furthermore, Tomavo [32] discovered that SAG3 also has a pivotal role in the invasion of *T. gondii* tachyzoites. SAG1 and SAG3 share 24% amino acid sequence identity, 12 cysteine residues and some identical short peptides [33]. These findings indicate that although *T. gondii* tachyzoites produce a variety of surface membrane proteins, the major ones share many similarities (e.g., sequence, function), suggesting that they may have evolved from a single ancestral protein. Nevertheless, interactions between these membrane proteins remain to be characterized.

### Relationship between *sag1* and strain virulence

To elucidate the relationship between *sag1* and *T. gondii* virulence, Howe et al. [34] examined the *sag1* locus from parasite lineages of varying virulence. Through digestion with three restriction enzymes and hybridization using specific nucleic acid probes, the P89, G622-M and ROD strains were found to share a restriction pattern with the *sag1*-1 allele, which is a hallmark of the RH strain. In addition, the restriction pattern of another allele in the locus, *sag1*-2, was different, and this allele has only been found in CEP, a strain that is widely recognized to have weak virulence. These results appeared to indicate that P89, G622-M and ROD were all virulent strains, although animal experiments showed that P89 and G622-M exhibited a similar virulence level to that of CEP; all three strains had the same 100% lethal dose (LD100) of 10,000 tachyzoites and the same LD50 of 100 tachyzoites. However, ROD, a recombinant strain, displayed a potent toxicity to mice with an LD_100_ of 100 tachyzoites and a 60% lethal dose of 10 tachyzoites [34]. It can therefore be concluded that *T. gondii* strains with the same *sag1* allele may still exhibit different virulence phenotypes, suggesting that the locus has a negligible effect on *T. gondii* virulence. These results can also be interpreted as these phenotypic differences being caused by the sequences flanking *sag1*. Hence, Howe et al. [34] used a 9-kb *Cla*I-*Eco*RI genomic fragment to probe pre-digested genomic DNA samples, and the subsequent Southern blot revealed that the RH strain displayed the same pattern as P89, G621-M and ROD and was different from that of CEP. This result appeared consistent with the virulence difference between the RH and CEP strains.

In addition, *Pvu*II digestion of the Southern blot samples revealed that both virulent strains, RH and ROD, had a *Pvu*II site, which was not found in either P89 or G622-M. Further experiments showed that the *Pvu*II site resides 1 kb upstream of the *sag1* locus, which was afterwards also corroborated by Lekutis et al. [35]. In the latter study, Lekutis et al. used the gene for hypoxanthine-xanthine-guanine phosphoribosyltransferase to generate a mutant lacking *sag1* by homologous recombination into the *T. gondii* genome. With the extension of *in vitro* culture time, the mutant displayed an invasive capacity 2-fold higher than that of the wild-type RH strain. However, when parasites were intraperitoneally inoculated into Swiss Webster mice, the mutant-infected group had a delayed acute-phase death compared to the RH-inoculated animals, indicating that the mutant was less virulent than the wild-type. Taken together, studies have indicated that the presence of allele 1 at the *sag1* locus is not alone sufficient to confer acute virulence, which is dictated by the *sag1* gene *per se* as well as by its flanking regions, especially the upstream sequences.

### Studies on SAG1 epitopes

A protein antigen does not require its intact molecule to generate antigenic effects; rather, the molecule produces the specific effects via individual epitopes [36]. Moreover, a protein antigen not only contains epitope structures closely related to immune recognition by B cells, T cells and NK cells but also harbors some elements that are unfavorable for protective immunity. As such, to improve the protective traits of a protein antigen, choosing proper epitope levels is necessary to eliminate deleterious components and retain advantageous elements [36]. Such studies on antigen epitopes of SAG1 have been conducted [11,25,37-41].

Nam et al. [25] reported that the antigenicity of SAG1 is primarily controlled by a 1/3 to 4/9 region from the N-terminus. Godard et al. used a mouse model in conjunction with sequence prediction and peptide scanning to identify 3 antigenic peptides (i.e., 48–67 aa, 238–256 aa and 279–285 aa). They concluded that the C-terminus was the dominant antigenic and immunogenic region and that the 238–256 aa peptide was also an important T cell epitope [11]. Begheto et al. used sera from toxoplasmosis patients to screen a cDNA phage display library of *T. gondii* and discovered that the C-terminus of SAG1 displayed stronger reactivity to the sera [37]. Likewise, Siachoque et al. examined the B cell epitopes of SAG1 and revealed that they were primarily concentrated near the C-terminus using a mouse model and peptide scanning [38]. Furthermore, Cardona et al. [40] used peptide scanning to investigate the B cell epitopes of SAG1 and discovered that in addition to several C-terminal fragments (181–200 aa, 241–260 aa, 261–280 aa and 301–320 aa), the sera of *T. gondii*-infected individuals recognized an N-terminal fragment (61–80 aa), although the 301–320 aa had the highest reactivity. Similarly, our laboratory used sera from *T. gondii*-infected pigs to screen B cell epitopes of SAG1 and showed that fragments composed of 91–120 aa, 151–180 aa, 271–300 aa and 301–336 aa were all specifically recognized by the porcine sera and that the 271–300 aa fragment displayed the strongest reactivity [41]. A comprehensive analysis of the above findings indicated that the sera from *T. gondii*-infected humans and pigs and SAG1-immunized mice exhibited some differences in serum recognition patterns. Fine mapping of antigenic epitopes could advance the development of diagnostic reagents and epitope vaccines for *T. gondii*.

### Mechanisms of the immune responses induced by SAG1

The SAG1 protein is abundant in tachyzoites and is highly immunogenic. Therefore, this protein can stimulate the host to generate relevant humoral and cellular immunity. Humoral immunity plays a role in countering *T. gondii* infection, but cellular immunity is the major mechanism whereby SAG1 induces the host to generate immune protection. This process requires different types of T lymphocytes (e.g., CD4^+^, CD8^+^ T cells) coordinated by many cytokines, including IFN-γ, IL-2 and TNF-a, and plays a crucial role in host anti-*T. gondii* protective immunity [42]. CD8^+^ T cells are the primary cell subpopulation that acts against *T. gondii* infection of target cells, and their cytotoxicity is antigen-specific. Once immunized, mice displayed elevated SAG1-specific CD8^+^ T cells and acquired an almost complete ability to counter acute *T. gondii* infection. Although CD8^+^ T cells do not require the assistance of CD4^+^ T cells to exert an immune protective effect, the presence of IFN-γ is essential for this event, and the anti-*T. gondii* function of CD8^+^ T cells is unaffected by the removal of CD4^+^ T cells but disappears without IFN-γ. In addition to CD8^+^ T cells, activated macrophages also participate in a host’s anti-*T. gondii* activities [43].

### Vaccines based on SAG1 of *T. gondii*

#### Gene deletion attenuated vaccine

Increased knowledge of *T. gondii* at the molecular level, greatly facilitated by the completion of the genome project, in combination with rapid progress in the development of genetic tools to manipulate the parasite, has generated opportunities to create new attenuated vaccines [44]. SAG1 of *Toxoplasma* has also been targeted for deletion and two types of SAG1 mutants have been generated. One was made by chemical mutagenesis and the other was recently genetically engineered (∆*sag1*). Both attach to, enter, and proliferate at approximately normal rates within host cells [28,45,46]. ∆*sag1* tachyzoites were lethal in susceptible C57BL/6 mice (although survival was slightly prolonged compared with wild-type infected mice), but deletion of *sag1* prevented an acute ileitis when tachyzoites were directly injected into the intestine. At present, the infectivity of *Toxoplasma* mutants that have been generated by reverse genetics has only been analyzed in mice and not in larger animals. In past years, various *Toxoplasma* gene deletion mutants have been generated – although the objective has usually been to gain further insight into the function of a particular gene rather than to generate a vaccine. However, it is likely that any biologically important gene will contribute to the fitness and/or virulence of the parasite, and consequently the vaccine potential of these mutants has been of significant interest.

### Subunit vaccines

Subunit vaccines have the advantage that specific immunogenic antigens are presented without adding antigens that are at best irrelevant and at worst capable of inducing febrile or disease-exacerbating immune responses. SAG1 vaccines for *T. gondii* have been studied extensively with promising results. To produce the vaccine, SAG1 antigen purified via a monoclonal antibody is used to immunize mice [13] to induce cytotoxicity of CD8+ cells, thereby generating high levels of IFN-γ and IL-2 and a protective immunity that combats acute and chronic infection. Due to the high cost of purifying tachyzoite membranes, molecular cloning has replaced this technique. Makioka et al. [47] PCR amplified the *SAG1* gene, cloned it into several vectors, including pTV118N, pKK233-2 and pGEX-1, and transformed it into several *E. coli* strains (JM109, JM105, etc.). Among these combinations, the pGEX-1 recombinant plasmid achieved a high expression level using a GST (glutathione S-transferase)-fusion protein. This recombinant protein has been demonstrated to be able to activate macrophages in animal experiments and to exhibit toxoplasmacidal effects in *in vitro* assays. More importantly, macrophage activation was shown to be caused by SAG1, not the GST tag, in the fusion protein. In addition, SAG1, adjuvant withstanding, has induced comparatively good protection in terms of limiting maternofetal transmission [48].

Although subunit vaccines are safer than inactivated whole-cell or live attenuated vaccines, they have weak immunogenicity and are not effectively recognized and presented by antigen-presenting cells. Hence, development of subunit vaccines for *T. gondii* requires not only antigen optimization but also adjuvants to boost immunogenicity and induce generation of immune-active compounds. With optimized antigens in combination with effective adjuvants, the resulting vaccines can modulate the intensity and types of immune responses and produce effective and long-lasting protective immunity [49].

### Bacterial and viral vector vaccines

Live bacterial vaccine vectors have been extensively used to deliver and express heterologous vaccine antigens to protect against cancer and various infectious agents, including AIDS. Live bacterial vaccines have the advantage that they can express multiple antigens, are easily mass produced, can be orally or intranasally applied, and induce strong immune responses. However, relatively few studies have tested whether heterologous expression of parasitic antigens with bacterial vaccine vector strains can lead to protective immunity. For example, oral immunization with a live attenuated *Salmonella typhimurium* vaccine strain was used to protect mice against *T. gondii*[50]. SAG1 and SAG2 were delivered with a eukaryotic expression plasmid which also contained cholera toxin (CT) subunits A2 and B. CT is known to have an adjuvant effect, and indeed the addition of CT subunits A2 and B induced a strong cellular immune response, as measured by induced specific IgG2a titers, splenocyte proliferation, and IFN-γ production. Upon challenge with 1000 RH tachyzoites the vaccinated mice survived longer, and 40 percent of the mice survived the whole trial period. Whether this vaccine could prevent tissue cyst formation and reduce cyst burden is unknown.

Although viral delivery of immunogenic antigens has been tested widely against various cancers and infectious diseases, including malaria, only a few studies have used this technique to express *Toxoplasma* antigens [44,51]. Vaccinations with viral vectors carrying *Toxoplasma* antigens have met with limited success. Lack of protection is either due to an improper immune response, and/or because non-protective antigens have been expressed.

### Nucleic acid vaccines

Nucleic acid vaccines were developed in the 1990s and are the third generation of vaccine after pathogen vaccines and subunit vaccines. DNA vaccines are simple to prepare and are low-cost. Thus, they are conducive to large-scale industrial production. These molecules are expressed in the eukaryotic environments of host cells; therefore, the products are similar to the conformation of the natural counterparts and are able to induce more effective immune responses against the target protozoan [52]. In addition, persistent immune responses also simplify the immunization procedure. A Japanese group attempted an intradermal injection using a gene gun to introduce a SAG1-encoding plasmid into mice then used the skin from the inoculation site to perform an allograft. The results demonstrated that the inoculated skin could stimulate the body to generate protective antigens that effectively prevented a lethal *T. gondii* infection [53]. Likewise, Nielsen et al. [54] immunized animals with the p1tpA-SAG1 plasmid, which yielded an 80-100% survival rate for RH-infected animals, whereas the control group, treated with the empty p1tpA vector, displayed a 20% survival rate, showing that direct immunization with the pltpA-SAG1 plasmid can generate remarkable protection against infection. Nucleic acid vaccines promote not only the generation of humoral and cellular immune cascades but also the induction of local immune responses and immune memory [55]. In addition, nucleic acid vaccine vectors also contain non-coding immunostimulatory sequences that have an immunoadjuvant function and can act on various types of immune cells to effectively generate Th1 responses [43].

### Multivalent vaccine

SAG1 is a stage-specific protein in *T. gondii* that is only present in tachyzoites but not bradyzoites. Monovalent SAG1 vaccines have been shown to yield only partial protection in test animals [56]. Hence, in the course of researching *T. gondii* vaccines, a consensus to develop multivalent vaccines that contain a combination of several antigens and target different developmental stages has been reached with the aim of overcoming the drawback of the monovalent candidate vaccines [57]. In this regard, SAG1 and ROP2 act synergistically during parasite invasion of host cells. As a consequence, the corresponding antibodies significantly block the infection of *T. gondii*. Alberto et al. immunized mice with a mixture of the pcDNA3/SAG1 and pcDNA3/ROP2 plasmids, which produced a Th1 immune response, proliferation of specific T cells, and a high IFN-γ titer. Likewise, immunization with a plasmid mixture was shown to induce enduring protection against infection with the RH strain, whereas the control cohort treated with a monovalent gene vaccine was not resistant to the infection [58].

Extracting and purifying a combination gene vaccine is a complicated process that is worsened by the fact that eukaryotic cells accept a limited quantity of foreign DNA. Furthermore, expression of foreign genes from several plasmids inevitably causes competition in the host gene expression system. As such, complex gene vaccines have become a focus of research. The effectiveness of SAG1 DNA vaccines was generally enhanced by utilizing cocktail vaccines comprising of other antigens [9,58-63]. Additionally, we also support the view that relatively short peptides containing B- and T-cell determinants can be highly immunogenic, and therefore may be considered suitable alternatives in subunit vaccine design. In our laboratory, we investigate murine immune responses to one linear B-cell epitope (derived from conserved regions of SAG1) when conjugated to two other defined T-cell epitopes (from conserved regions of GRA1 and GRA4) in a MAP arrangement. Immunization of BALB/c and Kunming mice with the MAP construct in Freund’s adjuvant induced not only a humoral immune response but also a cellular response. These responses were accompanied by significant levels of splenocyte proliferation and IFN-γ *in vitro*. After lethal challenge, vaccinated mice had increased survival time in comparison to unvaccinated controls [61].

Taken together, studies have indicated that immune protection of SAG1 is influenced by multiple factors. Adjuvants play an important role in the efficacy of immunizations. It is the general consensus of opinion that a type-1 response, particularly associated with CD8+ T cells producing IFN-γ, is the major mediator of immunity against *T. gondii* infection. Interestingly, vaccination with the Th2-inducing adjuvant ALUM incorporating SAG1 was able to promote survival of BALB/c mice infected with virulent parasites [14]. Other important factors in immunization experiments are the selection of animal model (species/strain) and the selection of *T. gondii* strain. For example, subcutaneous SAG1 vaccination protected guinea pigs [64] and BALB/c mice [48] against maternofetal transmission, but failed to protect CBA/J mice [48]. As mice are normally susceptible to *T. gondii* infection, parasite strains with low virulence or low doses of virulent strains may be preferentially selected by some researchers [65]. In some studies of immunization, oral challenges were made with cystogenic strains [66]. It is important to point out that unnatural routes of infection may be key determinants of mouse survival.

### Application of SAG1 in the diagnosis of toxoplasmosis

#### SAG1-based ELISA diagnosis

*T. gondii* is an opportunistic protozoan parasite. The isolation of the parasite by mouse bioassay is often difficult as the procedures are time-consuming, laborious and inefficient. As such, the serological approach is currently the most common diagnostic method [67-69]. Among various known antigens, SAG1 has potent immunogenicity and immune-reactivity; therefore, it is not only significant for vaccine research but is also widely applied in diagnostics [70-72].

The antigens used for ELISA detection are mainly parasite, excreted/secreted, recombinant or multi-epitope antigens. Parasite antigens and excreted/secreted antigens contain complex ingredients and are difficult to standardize quantitatively and qualitatively; therefore, the relevant diagnostic systems tend to vary in detection efficiency [73-76]. SAG1 is a main surface antigen of *T. gondii* that induces high titers of IgG, IgM and IgA despite only accounting for 3-5% of total cellular proteins [19]. In addition, this protein is also a tachyzoite-specific antigen and is highly conserved [22]. Thus, it is important for diagnostic purposes [38,77]. Several groups have attempted to use recombinant DNA technology to prepare *T. gondii* SAG1 recombinant antigens, most of which, unfortunately, produce insoluble forms and lose antigenicity due to misfolding. Although the material can be refolded to partially recover the antigenicity, the procedure is extremely inefficient (5-8%) and time consuming [78]. To address this issue, Wu et al. [79] tried different expression vectors and optimized expression conditions to express a truncated *SAG1* gene and obtained soluble recombinant protein, thereby establishing an ELISA diagnostic based on recombinant SAG1. This ELISA kit could be used to detect *T. gondii* infection sensitively and specifically. By comparison with the gold standard (the Western blot), the sensitivity and specificity of this ELISA kit is 93.9% and 100%, respectively, whereas the efficiency is also better than the 2 commercially available ELISA kits constructed from the whole parasite extracts. The results confirmed that the recombinant SAG1 protein is an ideal diagnostic antigen for the development of diagnostic kits for human toxoplasmosis. However, the exact composition and association of recombinant antigens to be used in immunoassays to detect *Toxoplasma* antibodies are still open questions. Using the B-cell epitopes of those antigens for the serodiagnosis of toxoplasmosis has been found to present several advantages, such as the precise knowledge of the composition of the diagnostic antigen, the ability to use more than one identified B-cell epitope, and easy standardization of the method. The multiepitope chimeric antigen including SAG1 is one of the most promising antigens for the development of diagnostic kits for routine toxoplasmosis screening [80]. Of course, the application of multi-epitope antigens is largely dependent on identifying antigenic epitopes and solving technical details involved in their design. These issues include identifying B cell epitopes and T cell epitopes, identifying optimal combinations of different B cell epitopes or B cell and T cell epitopes and avoiding new antigenic epitopes.

### *SAG1*-based molecular diagnostic methods

#### PCR

Because PCR sensitivity is closely linked to the copy number of the amplified gene, the *B1* gene, which has 35 copies and 200-300 repeats of a 520 bp fragment, is often used as a target gene [81-85]. However, the sensitivity of PCR is not only affected by the genomic copy number of the target gene but is also affected by the infection stage. Contini et al. [86] argued that for HIV patients, especially those that have undergone anti-*T. gondii* treatment, PCR targeting the *B1* gene is often negative. In contrast, PCR detection using primers specific to several target genes including *sag1, sag4* and *mag1* is often very effective. Savva et al. [87] first used PCR detection based on *SAG1*-specific primers and showed that they were able to amplify DNA from all of the seven test *T. gondii* strains but produced no bands from other parasites or microbes. Hence, these primers are highly specific. Hollman et al. extracted DNA from heart transplant biopsy specimens of 15 antibody positive subjects, and the DNA samples were used to amplify *sag1*, which identified 8 samples as positive [88]. In comparison, histology only identified 2 samples as positive, indicating that *sag1*-based PCR is clearly better than a histological approach.

### Loop-mediated isothermal amplification (LAMP)

LAMP is a simple technique that rapidly amplifies specific DNA sequences with high sensitivity under isothermal conditions [89]. LAMP products can easily be detected by the naked eye due to the formation of magnesium pyrophosphate, a turbid white by-product of DNA amplification that accumulates as the reaction progresses. In addition, LAMP products can be detected by direct fluorescence. Other fluorescent dyes, such as ethidium bromide, SYBR green and Evagreen, have also been used for visualization of LAMP products. Furthermore, Thekisoe et al. have reported that LAMP reagents are relatively stable even when stored at 25 or 37°C, which supports the use of LAMP in field conditions and resource-poor laboratories [90]. Recently, LAMP assays targeting the *sag1* gene have been developed, and these methods were found to be powerful diagnostic tools [91-93].

## Conclusions

SAG1 has been a focus of study because it is among the first few proteins that interact with host cells and is primarily involved in adhesion, signal transduction, invasion, material transport and host immune responses. Thus, this protein may be crucial for both the diagnosis of *T. gondii* infection and the ability to immunize against this parasite. This exciting progress on SAG1 will lay a solid foundation to combat toxoplasmosis.

## Competing interests

The authors declare that they have no competing interests.

## Authors’ contributions

YHW and HY drafted the manuscript. Both authors read and approved the final manuscript.
